# *per*-Alkoxy-pillar[5]arenes as Electron Donors: Electrochemical Properties of Dimethoxy-Pillar[5]arene and Its Corresponding Rotaxane

**DOI:** 10.3390/molecules25071627

**Published:** 2020-04-02

**Authors:** Nicholas Pearce, E. Stephen Davies, Neil R. Champness

**Affiliations:** School of Chemistry, University of Nottingham, University Park, Nottingham NG7 2RD, UK; Nicholas.Pearce1@nottingham.ac.uk (N.P.); Stephen.Davies@nottingham.ac.uk (E.S.D.)

**Keywords:** pillararene, rotaxane, electrochemistry, voltammetry, oxidation

## Abstract

1,4-dimethoxypillar[5]arene undergoes reversible multielectron oxidations forming stable radical cations, a property retained when incorporated in [2]rotaxanes, suggesting that pillar[5]arenes can be employed as viable, yet unreported, electron donors.

## 1. Introduction

Pillararenes have attracted much attention as macrocyclic hosts since their discovery by Ogoshi [1]. This interest is owed partly due to their symmetrical pillar-like shape that distinguishes them from other macrocycles such as calixarenes and cucurbiturils. The most common member of the pillararene family, pillar[5]arene, contains five 1,4-dialkoxybenzene subunits bridged by methylene spacers. Each rim of the tube-shaped macrocycle is lined with five oxygen atoms, which results in an electron-rich cavity from which the host properties arise, leading to the use of pillar[5]arenes in the construction of inclusion complexes [2,3,4,5,6,7], rotaxanes [8,9,10,11,12,13,14,15] and other mechanically interlocked systems [16,17,18,19,20].

The scope of pillararenes as hosts can be expanded by substituting the rim alkoxy positions with a desired group; either by cocyclisation of a functionalised monomeric subunit with 1,4-dimethoxybenzene [21,22]; by dealkylation and further reaction of the already formed macrocycle [23,24,25]; or by preorienting the cyclisation reaction with a 2,5-substituted benzyl alcohol [26,27], the latter affording ‘’rim-differentiated’’ pillararenes where each rim is pre-functionalised with a desired group. The chemistry of these fascinating molecules has recently been extended to a new family of tiara[5]arenes that exhibit more conformational flexibility [28].

The oxidation, or partial oxidation, of the alkoxybenzene subunits to the corresponding benzoquinones offers an alternative approach to modifying pillararene functionality [29,30,31,32,33,34]. When only some of the subunits are oxidised, a pillar[n]arene[5-n]quinone is prepared. Investigations into the voltammetric behaviour of these pillararene quinones have been reported [32,33], but have focussed specifically on the cathodic properties of the quinone containing macrocycle. In the case of pillar[5]quinone, Cheng and Kaifer [33] concluded that electrons are gained in a 2-1-2 uptake sequence, reflecting the molecule’s pentagonal symmetry. Similar results were reported by Saba and coworkers [32] for a series of 1,4-dimethoxypillar[n]arene[5-n]quinones, where the number of electron uptake processes increased with the number of quinone subunits, due to a concomitant increase in coulombic interactions. The electrochemistry of the fully dealkylated pentahydroquinone pillar[5]arene species has also been investigated [34]. We note that none of these studies have investigated the behaviour of the fully alkylated pillar[5]arene in which formation of a quinoidal group is prohibited upon oxidation.

## 2. Results and Discussion

**Scheme 1:**
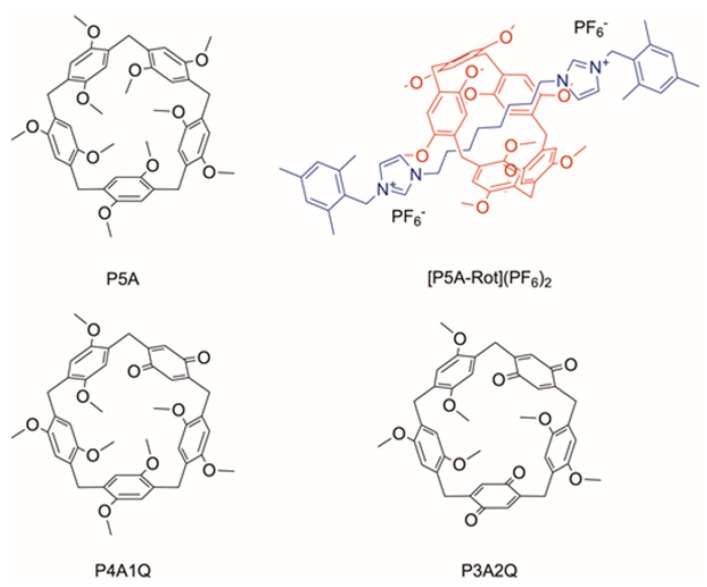
Molecules investigated in this study.

**Figure 1:**
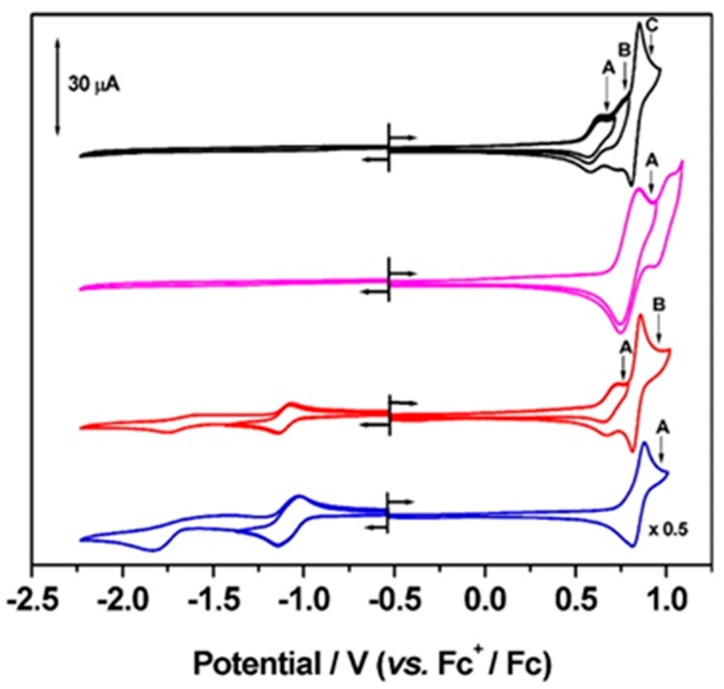
Cyclic voltammetry of P5A (black trace), [P5A-Rot]^2+^ (magenta trace), P4A1Q (red trace) and P3A2Q (blue trace), in CH_2_Cl_2_ containing [^n^Bu_4_N][BF_4_] (0.4 M) as supporting electrolyte, at ambient temperature and at 0.10 Vs^−1^. Letters indicate potentials applied in bulk electrolysis and spectroelectrochemical experiments; first oxidation (**A**), second oxidation (**B**) and third oxidation (**C**).

**Figure 2:**
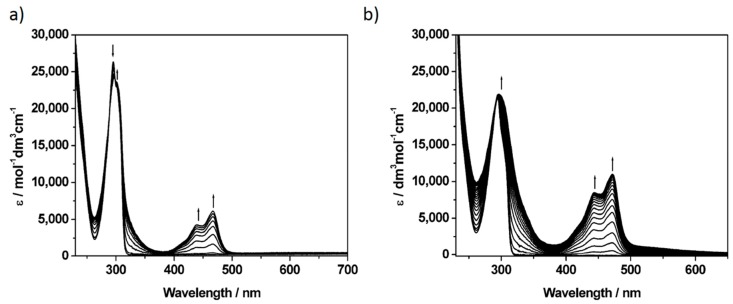
UV-vis absorption spectra showing (**a**) the inter-conversion of redox states in P5A between P5A and [P5A]+ (potential A) (**b**) the inter-conversion of redox states in [P5A-Rot]^2+^ upon oxidation at potential A. See Figure 1 for potentials applied. Spectra were recorded in CH_2_Cl_2_ containing [^n^Bu_4_N][BF_4_] (0.4 M) as the supporting electrolyte at 273 K.

Thus, we investigated the redox behaviour of 1,4-dimethoxypillar[5]arene (P5A, Scheme 1), the simplest fully alklyated pillar[5]arene, by cyclic voltammetry in CH_2_Cl_2_ solution (Figure 1; Table 1). For P5A, no redox processes were detected at negative potentials. At positive potentials, a series of oxidations were observed corresponding to the loss of multiple electrons from the P5A molecule. Interestingly, the first of these processes, at E ½ 0.61 V (vs. Fc^+^/Fc), shows reversible behaviour under the conditions of the experiment, and coulometry revealed this process to be a one-electron oxidation. Subsequent oxidation processes exhibit larger currents and scan rate dependence, with small peak separations (Figure S1), and diminished currents observed in the second half cycle of the CV scan, but surprisingly these processes all appear chemically reversible based on our UV/vis spectroelectrochemistry experiments (Figure 2 and Figure S3; Table 2).

In the neutral state, the absorbance spectrum of P5A contains a sharp peak at 295 nm, attributed to the nonconjugated dimethoxybenzene subunits, with no features at lower energy. When a potential of +0.67 V (A in Figure 1) was applied to a solution of P5A in CH_2_Cl_2_, containing [Bu_4_N][BF_4_] as supporting electrolyte, oxidation resulted in new, lower energy, absorbance bands at 467 and 439 nm. The original absorbance band remained, shifting slightly to 297 nm, without undergoing any significant loss of intensity. The application of a reducing potential fully regenerated the spectrum of neutral P5A. Thus, the use of dry aprotic solvent avoids oxidation of dimethoxybenzene units to the corresponding quinone, which would have resulted in the irreversible loss of the attached methyl groups and the generation of bands characteristic of a quinone derivative (Figures S6–S8). The application of larger positive potentials resulted in similar changes to the spectrum; the bands generated on first oxidation are increased in intensity but remain unshifted, indicating that a series of identical, isolated chromophores are generated upon oxidation.

**Figure 3:**
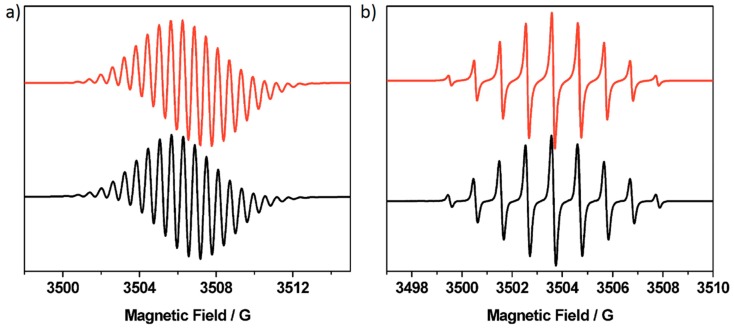
Experimental (black) and simulated (red) X-band EPR spectra of: (**a**) [P5A]^+^ and (**b**) [P4A1Q]^−^. Samples were electrogenerated from the parent molecules at 273 K as CH_2_Cl_2_ solutions containing [^n^Bu_4_N][BF_4_] (0.4 M) as the supporting electrolyte. Spectra were recorded at ambient temperature. Simulated spectra were generated using the parameters listed in Table 3.

To better understand the nature of these oxidations, and noting that the first of these is a one-electron process, an EPR spectrum was recorded for [P5A]^+^ (Figure 3; Table 3). The experimental spectrum was reproduced by simulation using a series of 2, 4 and 8 equivalent hydrogens with hyperfine coupling of 1.720, 1.136 and 0.570 × 10^−4^ cm^−1^, respectively. This simulation suggests that the unpaired electron resides around the core of a single arene subunit. This interaction is represented by the largest coupling; the unpaired electron also interacts with two equivalent protons on each of the methylene bridges, six equivalent protons from the two attached methoxy groups and similar small coupling from interactions with a single proton in each of the adjacent arene groups. 1,4-dimethoxybenzene, the constituent subunit of P5A, can also be oxidised electrochemically; however, EPR spectroscopic studies reveal the presence of two paramagnetic species [35]. Buck and Wagoner [36] were able to isolate and characterise a dimeric compound as the product of this electrochemical reaction indicating instability of the radical cation. In contrast, the [P5A]^+^ radical does not undergo rapid further reaction, suggesting that increased ring substitution and inclusion into a cyclic oligomer better protect the dimethoxybenzene monomers from radical degradation pathways [37,38,39].

We have also investigated the electrochemical properties of P5A when employed as a macrocyclic host in a simple rotaxane [P5A-Rot](PF_6_)_2_ (Scheme 1) [10]. Cyclic voltammetry of [P5A-Rot](PF_6_)_2_ (Figure 1; Table 1) shows apparently simplified electrochemistry relative to P5A with a reversible oxidation process at 0.80 V (vs. Fc^+^/Fc), shifted ca. 0.2 V more positive than that of the first oxidation in P5A. This change most likely results from the electrostatic interactions between P5A and the imidazolium moieties of the rotaxane, and demonstrates that occupation of the P5A cavity is readily detectable by electrochemistry.

Upon one-electron oxidation of [P5A-Rot](PF_6_)_2_, new absorbance bands develop at 472 and 443 nm, which are consistent with those observed for oxidised [P5A]^+^ (Figure 2b). Upon completing oxidation, reduction resulted in the loss of these bands and the regeneration of the characteristic 294 nm band of [P5A-Rot]^2+^ exactly, although the observation of an additional low intensity feature between 325 and 425 nm indicated that the oxidation process was not completely reversible under these conditions (Figure S5). The experimental EPR spectrum of oxidised [P5A-Rot]^3+^ (Figure S9) resembles that of [P5A]^+^, and was simulated using the same set of couplings albeit with slightly larger magnitudes.

To complement the study performed by Saba et al. [32], we also looked at the electrochemical properties of some partially oxidised variants of P5A in which one (P4A1Q) or two (P3A2Q) of the dimethoxybenzene monomers had been chemically oxidised to a quinone. The pillar[n]arene[5-n]quinone molecules were synthesised following the procedure reported by Xie and coworkers [31] in which a stoichiometric quantity of (NH_4_)_2_Ce(NO_3_)_6_ in CH_2_Cl_2_ is used as the oxidant. For P3A2Q, this method selectively generates the A,C constitutional isomer, simplifying purification. P4A1Q and P3A2Q each display a quinone-based reduction in their cyclic voltammetry (Figure 1; Table 1). In the case of P3A2Q, containing two quinone groups, this reduction appears broad, suggesting limited communication between the two quinone subunits. These processes were partially resolved into a peak and shoulder by square wave voltammetry (Figure S1; Table 4). Similar communication was also noted by Saba et al. in their voltammetry of P3A2Q in acetonitrile, where the effect was slightly more pronounced [32]. The reductions of P4A1Q and P3A2Q were followed by UV/vis spectroelectrochemistry (Figure 2, Figures S6–S8), and in each case the process was essentially reversible. Upon reduction, bands associated with the neutral species were replaced by two new bands, at ca. 325 nm and a broad band in the 400–500 nm region, and the intensity of these new bands in the reduced spectrum of P3A2Q was twice that found in reduced P4A1Q, consistent with generation of a common chromophore, based on the quinone, in a 2:1 ratio. EPR spectroscopy of electrochemically generated [P4A1Q]^−^ (Figure 3b) shows, by simulation of the fluid solution spectrum, coupling of the radical electron to two environments, containing two and four equivalent protons, consistent with a quinone based reduction and with interactions extending into the adjacent methylene bridges. A similar result was obtained for [P3A2Q]^2−^ (Figure S11), although the fluid solution spectrum showed greater line broadening and was superimposed over a broad baseline feature. Noting that the reduction of P3A2Q involves the addition of electrons to two quinone groups, we cooled the sample to 77 K and observed features consistent with spin-pairing between the added electrons forming a triplet state, as indicated by observation of the characteristic weak half-field signal (Figure S11).

The cyclic voltammetry of P4A1Q and P3A2Q exhibit significant differences upon oxidation. Whilst P4A1Q initially undergoes a one-electron oxidation, as determined by coulometry, that appears reversible, the corresponding process is absent from the cyclic voltammetry of P3A2Q and is replaced instead by a multielectron oxidation at a higher potential (Figure 1). For P3A2Q, the UV/vis spectroelectrochemistry showed poor reversibility in oxidation, therefore investigations were not continued (Figure S8). For P4A1Q, the UV/vis spectroelectrochemistry showed reasonable reversibility for the first oxidation; bands consistent with those observed in the oxidation of P5A were developed (Figure S6), indicating an arene-based chromophore. These features are lost upon reduction, and the 294 nm band, characteristic of the neutral molecule, is regenerated exactly, although a slight change in intensity was observed in the 261 and 269 nm bands. A similar trend was noted when investigating the second oxidation process, with changes in the 261 and 269 nm bands becoming more pronounced after the redox cycle (Figure S6). One-electron oxidised P4A1Q, [P4A1Q]^+^ was paramagnetic (g_iso_ 2.0036); however, the fluid solution EPR spectrum was insufficiently resolved to allow further analysis (Figure S12).

The behaviour of the pillararene-quinones as macrocyclic hosts was also probed by attempting to synthesise a rotaxane analogous to P5A-Rot with P4A1Q. First, a ‘’capping’’ approach was employed, replicating the synthesis of (P5A-Rot)^2+^, although only a negligible amount of the rotaxane could be generated in this manner. An NMR investigation by Han et al. using an alkylammonium guest indicated no complexation with P4A1Q, probably due to electrostatic repulsion between the quinone and the alkylammonium moieties [32]. Thus, we attempted synthesis of a P4A1Q rotaxane by chemical oxidation. Reaction between (NH_4_)_2_Ce(NO_3_)_6_ and (P5A-Rot)^2+^ also proved to be unsuccessful: although some P4A1Q was generated, dissociated from the diimidazolium axle. The majority of the rotaxane was unchanged after the reaction indicating the presence of a guest molecule within the macrocyclic cavity is unfavourable to the quinone forming reaction. These investigations indicate that replacement of even one of the dimethoxybenzene subunits within the pillararene macrocycle with a quinoidal group is sufficient to alter the host properties for such rotaxane forming reactions.

## 3. Conclusions

Overall, we have established that pillar[5]arene undergoes a reversible one-electron oxidation process to form a stable radical in dry aprotic solvent. Due to the unique shape of the macrocycle, these radicals are less prone to oxidative degradation or polymerisation than observed for free 1,4-dimethoxybenzene. The oxidative properties of the pillar[5]arene molecule, although still present, are changed by the formation of inclusion complexes as a result of electrostatic intermolecular interactions between host and guest. Studies with mixed pillararene-quinone macrocycles reveal that these oxidations are retained alongside a new quinone-based reduction, forming a compact, cyclic donor-acceptor dyad. Our results indicate that pillar[5]arene could serve as a suitable electron donor in the formation of charge-transfer compounds with an appropriate electron-accepting guest paving the way to more sophisticated supramolecular organic electronic systems.

## Figures and Tables

**Table 1:** Cyclic voltammetric data ^a^.

Compound	1^st^ Oxidation E_1/2_/V	2^nd^ Oxidation E_1/2_/V	Reduction	ΔE Fc^+^/Fc /V
P5A	+0.61 (0.08)	+0.83 (0.04) ^b,c^		(0.07)
[P5A-Rot](PF_6_)_2_	+0.80 (0.10)	-		(0.07)
P4A1Q	+0.69 (0.08)	+0.84 (0.04) ^c^	−1.11 (0.07) ^d^	(0.07)
P3A2Q	+0.85 (0.07) ^c^	-	−1.08 (0.12) ^e,f^	(0.07)

^a^ CH_2_Cl_2_ containing [NBu_4_][BF_4_] (0.4 M) as supporting electrolyte, at ambient temperature. Potentials quoted at 0.10 Vs^−1^ against E_1/2_ Fc^+^/Fc used as the internal standard. Values in brackets are ΔE (= E_p_^a^ – E_p_^c^); ^b^ unresolved process between these oxidations; ^c^ shows significant scan rate dependence ^d^ second reduction at E_p_^c^ −1.72 V; ^e^ centre of two overlapping processes; ^f^ additional reduction at E_p_^c^ −1.72 V.

**Table 2:** UV/vis spectroscopic data. λ_abs_/nm (ε x 10^−3^/mol^−1^dm^3^cm^−1^) ^a.^

Compound	Neutral	1^st^ Oxidation	2^nd^ Oxidation	3^rd^ Oxidation	1^st^ Reduction
P5A	295 (2.6)	297 (2.4), 439 (0.4), 467 (0.6)	298 (2.4), 440 (0.7), 467 (0.9)	301 (2.4), 439 (1.0), 467 (1.2)	-
[P5A-Rot](PF_6_)_2_	294 (2.2)	296 (2.2), 443 (0.8), 472 (1.1)			
P4A1Q	261 (1.3), 269 (1.4), 294 (1.7)	258 (1.6), 266 (1.6), 296 (1.7), 440 (0.4), 467 (0.5)	259 (1.8), 299 (1.7), 440 (0.5), 466 (0.7)		295 (1.9), 325 (0.7), 436 (0.6)
P3A2Q	260 (2.2), 267 (2.1), 293 (1.3), 436 (0.1)	254 (4.1), 456 (0.7)			294 (1.5), 323 (1.4), 432 (1.0)

^a^ In CH_2_Cl_2_ containing [NBu_4_][BF_4_] (0.4 M) as supporting electrolyte, at 273 K.

**Table 3:** EPR spectroscopic data ^a^.

Compound	g_iso_	a_iso/_x 10^−4^ cm^−1^	Linewidth/G	Lineshape
[P5A]^+^	2.0038	1.720 (2H), 1.136 (4H), 0.570 (8H)	0.25	Gaussian
[P5A-Rot]^3+^	2.0037	1.780 (2H), 1.220 (4H), 0.580 (8H)	0.37	Lorentzian
[P4A1Q]^-^	2.0050	1.947 (2H), 0.958 (4H)	0.12	Lorentzian
[P4A1Q]^+^	2.0036			
[P3A2Q]^-^	2.0051	1.947 (2H), 0.958 (4H)	0.22	Lorentzian

^a^ In CH_2_Cl_2_ containing [^n^Bu_4_N][BF_4_] (0.4 M) as supporting electrolyte, at ambient temperature.

**Table 4:** Square Wave Voltammetric data ^a^.

Compound	1^st^ Oxidation /V	2^nd^ Oxidation /V	3^rd^ Oxidation /V	1^st^ Reduction /V	2^nd^ Reduction /V
P5A	+0.61	+0.75	+0.83	-	-
[P5A-Rot](PF_6_)_2_	+0.80	+0.98	-	-	-
P4A1Q	+0.69	+0.83	-	−1.10	−1.66
P3A2Q	+0.84^b^	-	-	−1.05^c^	−1.64

^a^ CH_2_Cl_2_ containing [^n^Bu_4_N][BF_4_] (0.4 M) as supporting electrolyte, at ambient temperature. Potentials reported against E_1/2_ Fc^+^/Fc; ^b^ unresolved shoulder at less positive potential; ^c^ unresolved shoulder at more negative potential.

## Supplementary Materials

The following are available online, Experimental details, Figure S1: Scan rate data for all compounds; Figure S2: Square wave voltammograms for all compounds; Figures S3 and S4: UV-vis absorbance spectra for P5A; Figure S5: UV-vis absorbance spectra for [P5A-Rot](PF_6_); Figures S6 and S7: UV-vis absorbance spectra for P4A1Q; Figure S8: UV-vis absorbance spectra for P3A2Q; Figure S9: EPR spectrum of [P5A-Rot]^3+^; Figures S10 and S11: EPR spectra of [P3A2Q]^2-^; Figure S12: EPR spectrum of [P4A1Q]^+^.
