# Supplementary material for: per-Alkoxy-pillar[5]arenes as Electron Donors: Electrochemical Properties of Dimethoxy-Pillar[5]arene and Its Corresponding Rotaxane
Source: Molecules. 2020 Apr 2;25(7):1627. doi: 10.3390/molecules25071627 (PMC7180461; doi:10.3390/molecules25071627)
Supplement: Supplementary file 1 [file molecules-25-01627-s001.pdf]

Supporting Information:

per-Alkoxy-pillar[5]arenes as Electron Donors: Electrochemical Properties of Dimethoxy-Pillar[5]arene and its Corresponding Rotaxane

Nicholas Pearce, E. Stephen Davies and Neil R. Champness

Supporting Information Table of Contents

Reagents and Synthesis Page 1

Electrochemical and Spectroelectrochemical Measurements Page 2

EPR spectroscopy Page 9

References Page 11

Reagents and Purification

All chemicals and solvents were purchased from Merck, Fluorochem or VWR. NMR spectra were recorded at room temperature using Bruker AV400 or AV(III)400 instruments. Deuterated solvents were used as specified. Chemical shifts were recorded referenced to solvent residue for ^{1}H and ^{13}C experiments. Mass spectra were recorded using a Bruker microTOF II. Column chromatography was performed on silica gel 60 Å. Ferrocene was purchased from Aldrich and used as received. [$^{n}Bu_4N$][BF_4] was synthesised from commercially available reagents.

P5A,[S1] P4A1Q[S2] and P3A2Q[S2] were synthesised according to literature procedures. [P5A-Rot](PF_6)$_2$ was synthesised by anion exchange from the iodide analogue [P5A-Rot](I)$_2$, described in detail below. The initial synthesis of [P5A-Rot](I)$_2$ has been reported previously.[S1]

Synthesis and Characterisation Data for P5A-Rot

[P5A-Rot](I)$_2$ (109 mg, 72 µmol) was dissolved in ethanol (20 mL) and heated to 70 °C. NH_4PF_6 (247 mg, 1.48 mmol) was added and the mixture stirred for 30 min before cooling to room temperature. The white product was collected by centrifugation, washing three times with methanol to remove excess NH_4PF_6, leaving the product as a white powder, 95 mg, 85%. ^{1}H NMR (400 MHz, $CDCl_3$) δ ppm 7.83 (s, 2 H), 7.03 (s, 4 H), 6.83 (s, 10 H), 6.59 - 6.66 (m, 4 H), 5.41 (d, *J* = 1.6 Hz, 4 H), 3.73 (s, 30 H), 3.72 (s, 10 H), 2.36 (s, 6 H), 2.36 (s, 12 H), 2.23 (t, *J* = 8.5 Hz, 4 H), 0.87 - 0.97 (m, 4 H), 0.53 - 0.66 (m, 4 H), 0.15 - 0.32 (m, 4 H). ^{13}C NMR (400 MHz, $CDCl_3$) δ ppm 150.93, 140.32, 137.98, 133.88, 130.11, 129.77, 124.81, 122.60, 119.49, 115.31, 57.24, 48.65, 47.35, 29.62, 29.07, 28.46, 26.60, 21.11, 19.42. ^{19}F NMR (376 MHz, $CDCl_3$) δ ppm -72.63 (d, *J* = 711 Hz, 12F). ^{31}P NMR (162 MHz, $CDCl_3$) δ ppm - 144.21 ppm (sept, *J* = 713 Hz, 2P). HRMS (ESI)$^{+}$ m/z found 631.3647, 1407.6907 ($C_{79}H_{98}N_4O_{10}$ $[M]^{2+}$ requires 631.3636, $C_{79}H_{98}F_6N_4O_{10}P$ $[M + PF_6]^{+}$ requires 1407.6919).

Electrochemical Methods

Cyclic Voltammetry

Cyclic voltammetry was carried out using an Autolab PGSTAT20 potentiostat under an argon atmosphere using a three-electrode arrangement in a single compartment cell. Glassy carbon was used as the working electrode, platinum wire as the secondary electrode and a saturated calomel reference electrode, chemically isolated from the test solution via a fritted bridge tube containing electrolyte solution, in the cell. An analyte concentration of 1 mM was used with [^{n}Bu$_4$N][BF$_4$] (400 mM) as a supporting electrolyte. Redox potentials are referenced to the ferrocenium/ferrocene couple, which was implemented as an internal reference. No compensation was applied for internal resistance.

Spectroelectrochemistry

UV/vis spectroelectrochemical measurements were performed using an optically transparent quartz electrochemical cell, with a 0.5 mm path length. A three-electrode configuration of a platinum/rhodium gauze working electrode, platinum wire secondary electrode and a silver/silver chloride reference electrode (chemically isolated via a fritted bridge tube) were used in the cell. The potential at the working electrode was regulated with a Sycopel Scientific Ltd DD10M potentiostat and the spectra recorded with a Perkin Elmer 16 spectrophotometer. Temperature control was achieved with a stream of chilled nitrogen gas (cooled by passing through a tube submerged in liquid nitrogen) across the surface of the cell, adjusting the flow rate as necessary in response to a temperature sensor (±0.1 °C). [^{n}Bu$_4$N][BF$_4$] (400 mM) was used as the supporting electrolyte for the experiments.

Bulk Electrolysis and Electron Paramagnetic Resonance Spectroscopy

Bulk electrolysis was performed under an argon atmosphere at 0 °C in a two-component cell: a platinum/rhodium gauze working electrode and secondary electrode are separated by a glass frit. A silver/silver chloride reference electrode was bridged to the test solution through a vycor frit, oriented at the centre of the working electrode. The working electrode compartment, containing analyte (1 mM), was stirred rapidly with a magnetic stirrer bar during electrolysis. [^{n}Bu$_4$N][BF$_4$] (400 mM) was used as the supporting electrolyte for the experiments. After electrolysis was completed, the prepared solution was transferred by cannula to a quartz EPR tube for analysis on a Bruker EMX spectrometer. Solution phase (fluid) spectra were recorded at room temperature Spectra were simulated when possible using WIN EPR SimFonia software.

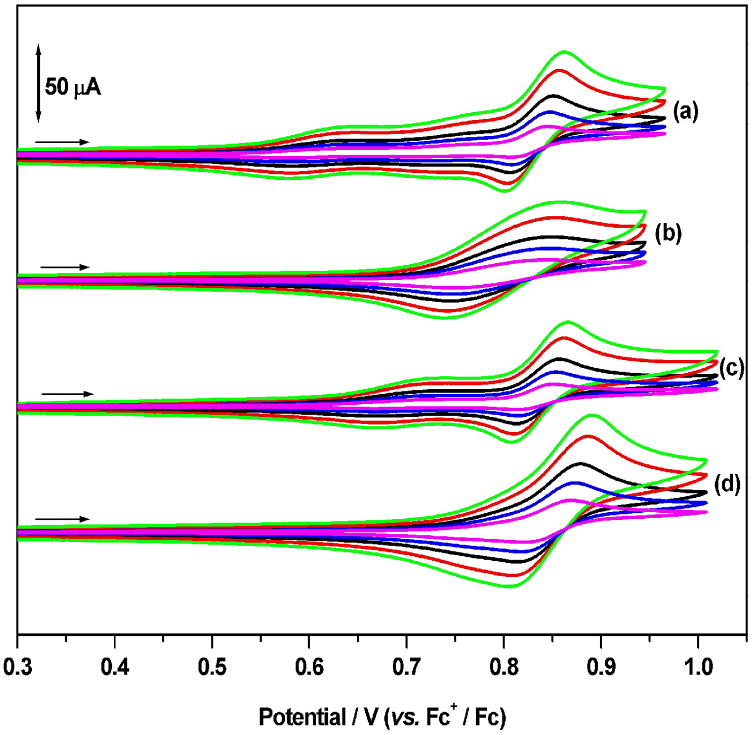

Figure S1. Effect of scan rate on the peak separations for (a) P5A, (b) [P5A-ROT]$(PF_6)_2$, (c) P4A1Q and (d) P3A2Q. Scan rates of 0.02, 0.05, 0.10, 0.20 and 0.30 Vs^{-1} are shown in magenta, blue, black, red and green, respectively.

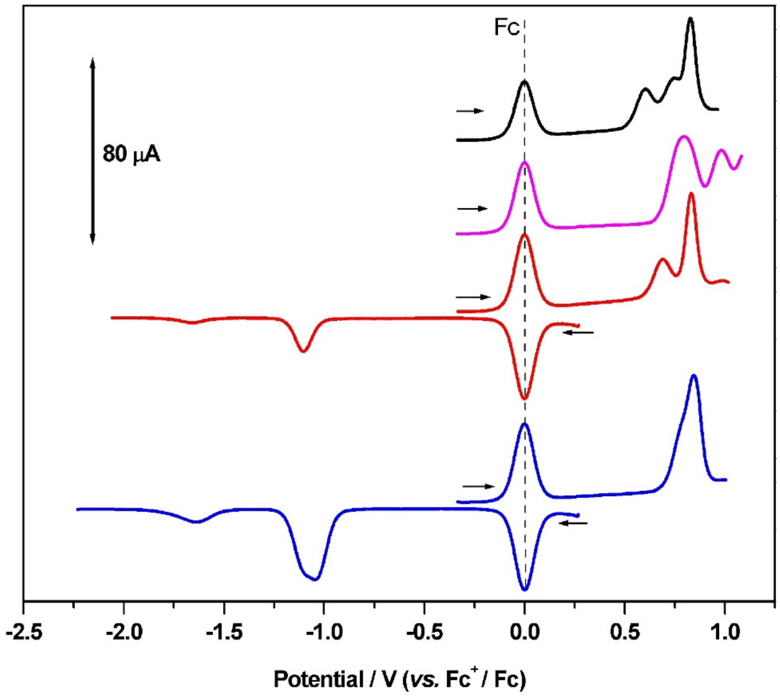

Figure S2 Square wave voltammetry of: P5A (black trace), [P5A-Rot]$(PF_6)_2$ (magenta trace), P4AQ1 (red traces) and P3AQ2 (blue traces). In CH_2Cl_2 containing [$^nBu_4N$][BF_4] (0.4 M) as supporting electrolyte, at ambient temperature.

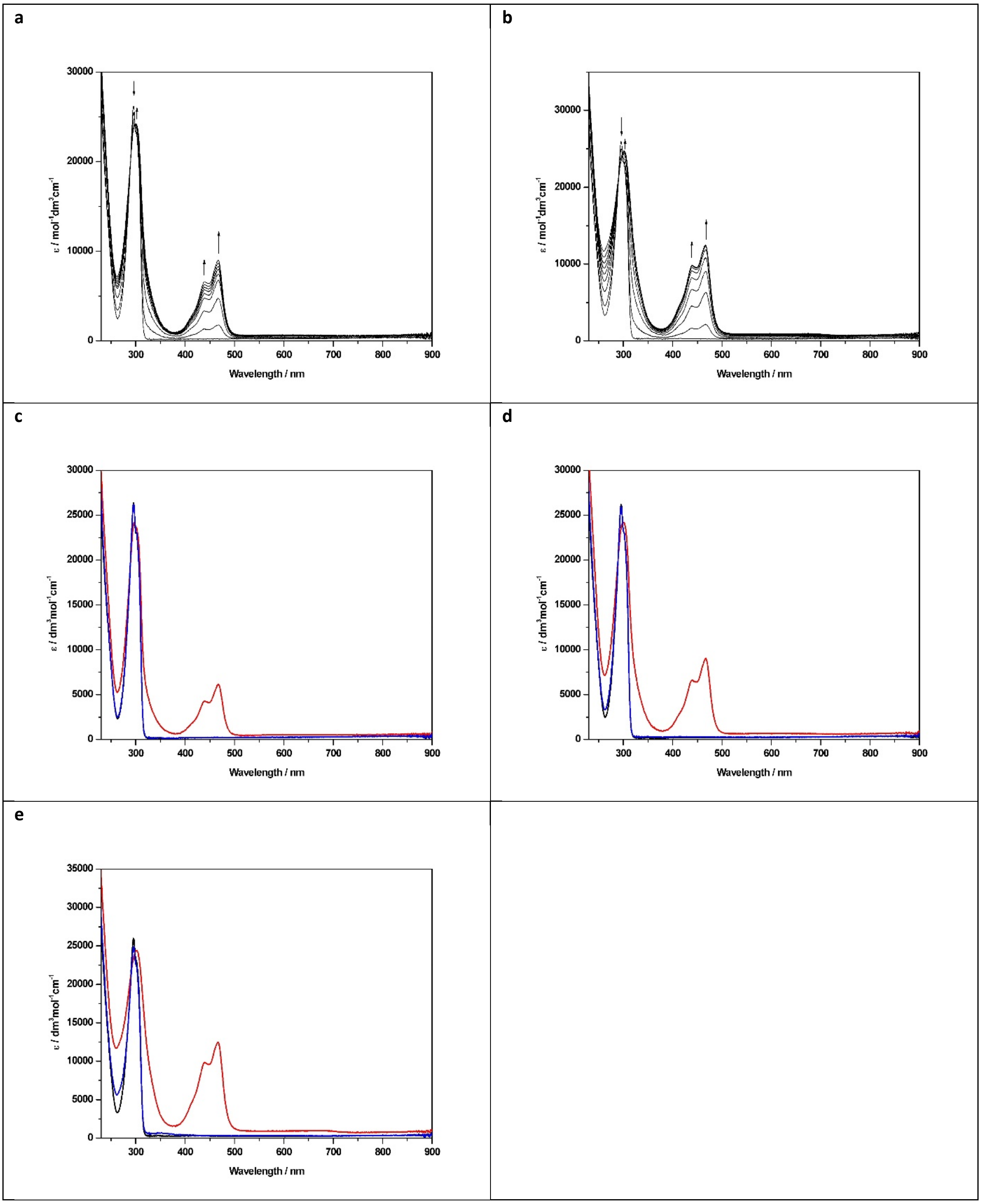

Figure S3 UV-vis absorption spectra showing: (a) second oxidation of P5A starting from the neutral state. Arrows indicate the progress of oxidation (b) third oxidation of P5A starting from the neutral state. Arrows indicate the progress of oxidation. (c) P5A before 1st oxidation (black trace), oxidised (red trace) and after the redox cycle (blue trace). (d) P5A before second oxidation (black trace), oxidised (red trace) and after the redox cycle to neutral state (blue trace). (e) P5A before third oxidation (black trace), oxidised (red trace) and after the redox cycle to neutral state (blue trace).Spectra were recorded in dichloromethane containing [$^{n}Bu_4N$][BF_4] (0.4 M) as the supporting electrolyte at 273 K.

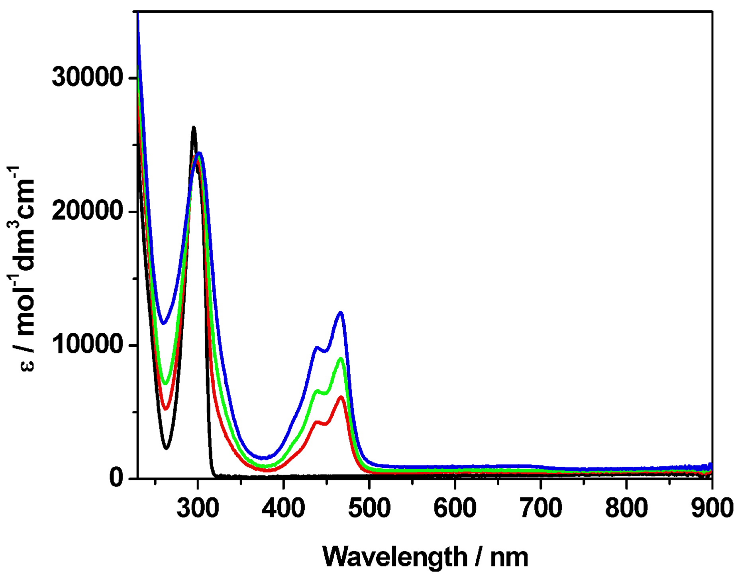

Figure S4 UV-vis absorption spectra showing the spectrum of P5A (black trace) and after oxidation at potential A (red trace), B (green trace) and C (blue trace). See Figure CV for potentials applied. Spectra were recorded in CH_2Cl_2 containing [$^nBu_4N$][BF_4] (0.4 M) as the supporting electrolyte at 273 K.

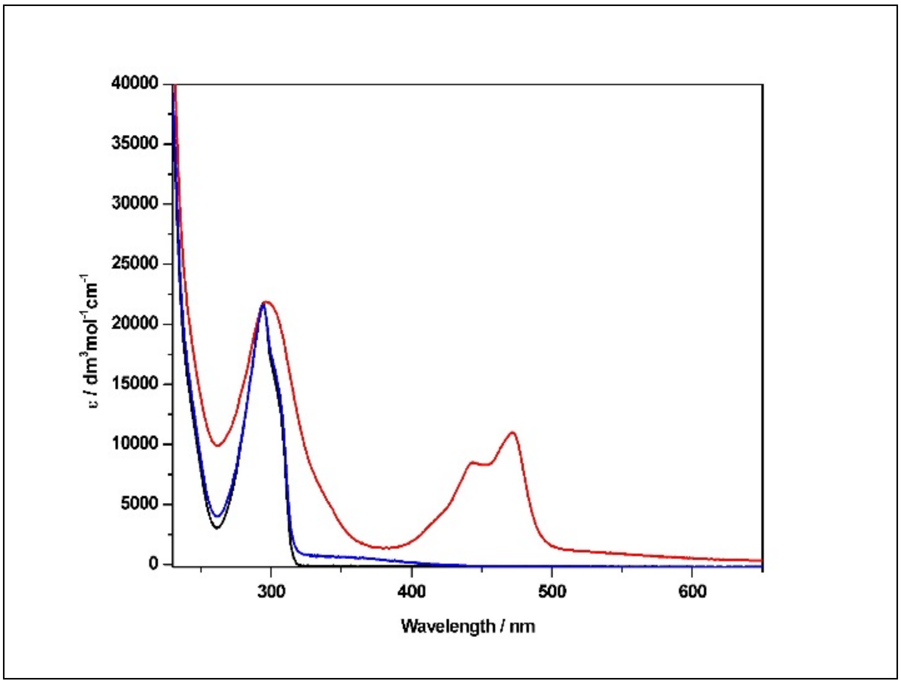

Figure S5 UV-vis absorption spectra showing [P5A-Rot](PF_6)$_2$ before oxidation (black trace), oxidised (red trace) and after the redox cycle (blue trace). Spectra were recorded in dichloromethane containing [$^nBu_4N$][BF_4] (0.4 M) as the supporting electrolyte at 273 K.

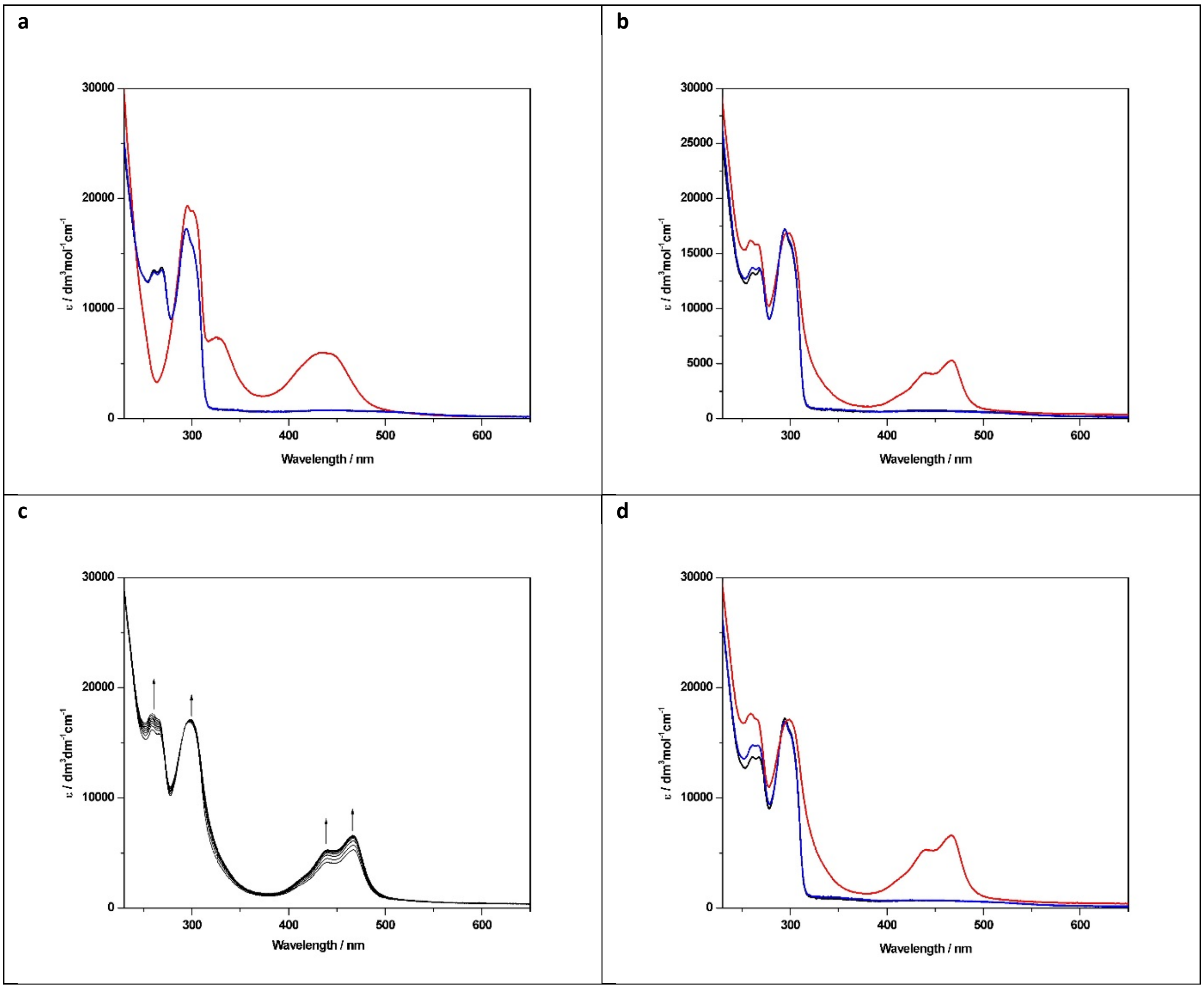

Figure S6 UV-vis absorption spectra showing: (a) P4A1Q before reduction (black trace), reduced (red trace) and after the redox cycle (blue trace) (b) P4A1Q before oxidation (black trace), oxidised (red trace) and after the redox cycle (blue trace) (c) second oxidation of P4A1Q. Arrows indicate the progress of oxidation (d) P4A1Q before second oxidation (black trace), oxidised (red trace) and after the redox cycle to neutral state (blue trace). Spectra were recorded in dichloromethane containing [$^{n}Bu_4N$][BF_4] (0.4 M) as the supporting electrolyte at 273 K.

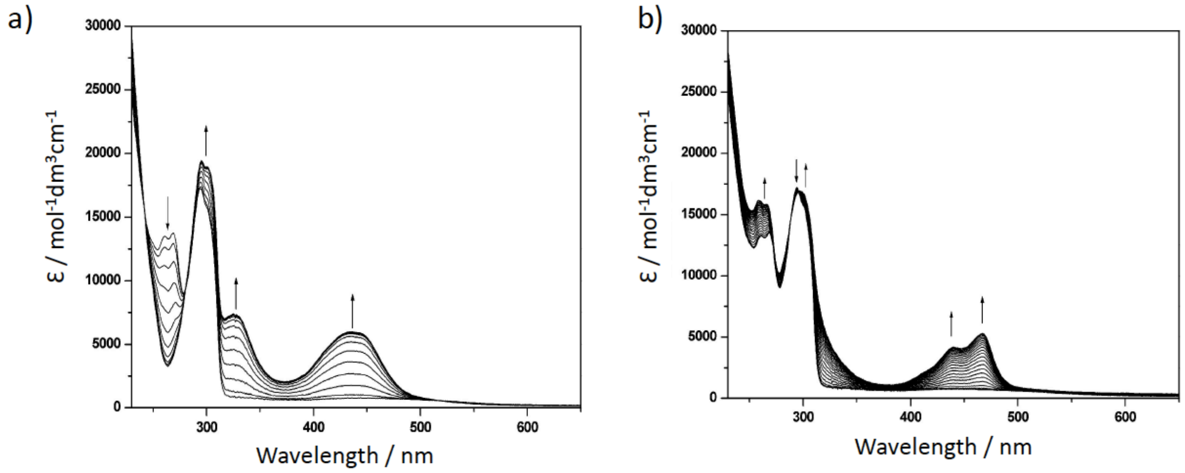

Figure S7 a) UV-vis absorption spectra showing: the inter-conversion of redox states in P4A1Q between P4A1Q and [P4A1Q]$^{+}$ (potential A); b) the inter-conversion of redox states in P4A1Q between P4A1Q and [P4A1Q]$^{-}$ (potential A). See Figure CV for potentials applied. Spectra were recorded in CH_2Cl_2 containing [$^{n}Bu_4N$][BF_4] (0.4 M) as the supporting electrolyte at 273 K.

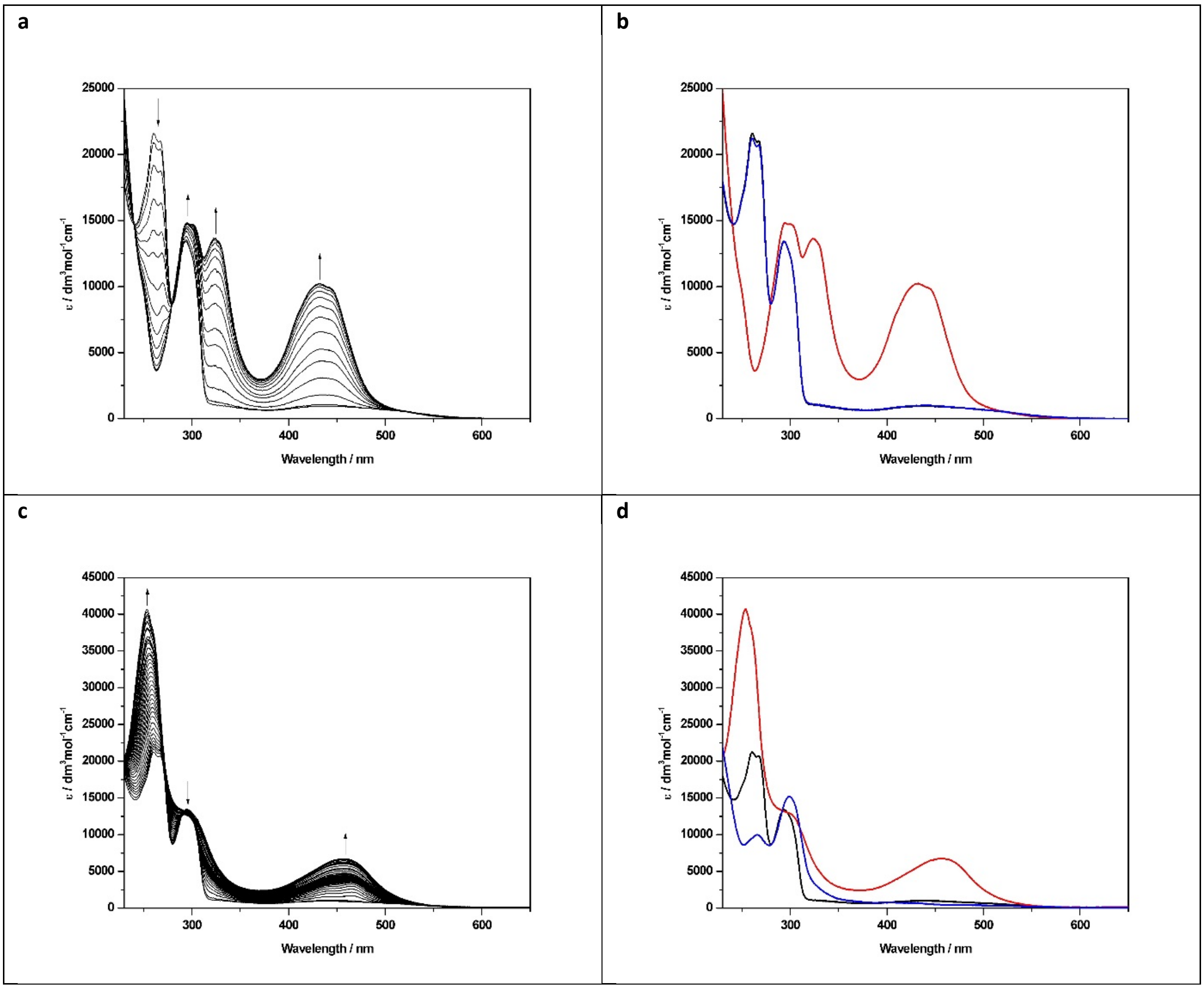

Figure S8 UV-vis absorption spectra showing: (a) reduction of P3A2Q. Arrows indicate the progress of reduction. (b) P3A2Q before reduction (black trace), reduced (red trace) and after the redox cycle (blue trace) (c) oxidation of P3A2Q. Arrows indicate the progress of oxidation (d) P3A2Q before oxidation (black trace), oxidised (red trace) and after the redox cycle (blue trace). Spectra were recorded in dichloromethane containing [$^{n}Bu_4N$][BF_4] (0.4 M) as the supporting electrolyte at 273 K.

EPR Spectroscopy

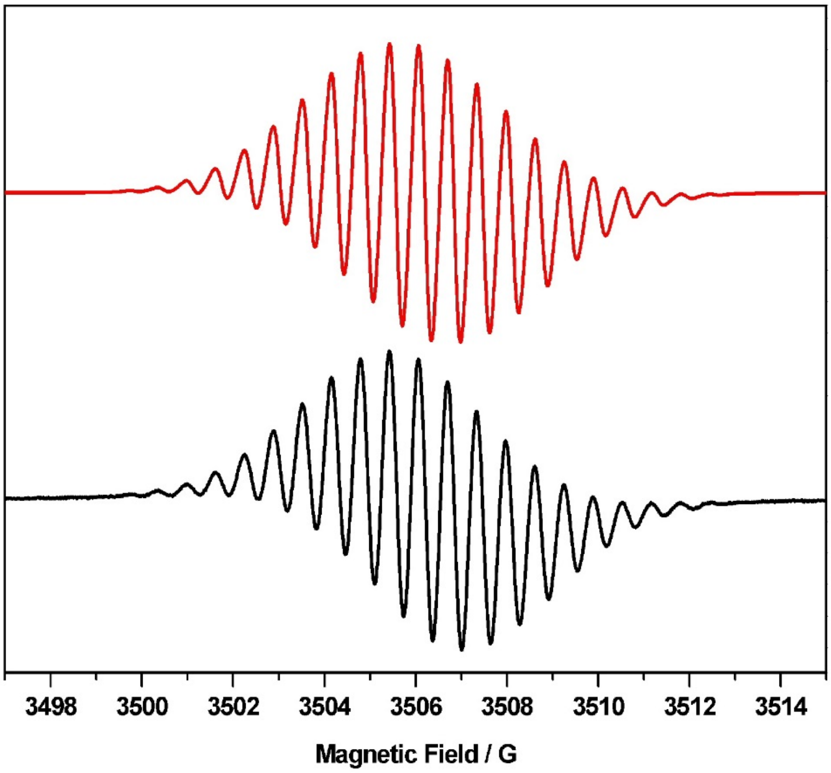

Figure S9 Fluid solution EPR spectrum of $[P5A\text{-}Rot]^{3+}$ as a solution in CH_2Cl_2 containing $[^nBu_4N][BF_4]$ (0.4 M) as supporting electrolyte, at ambient temperature.

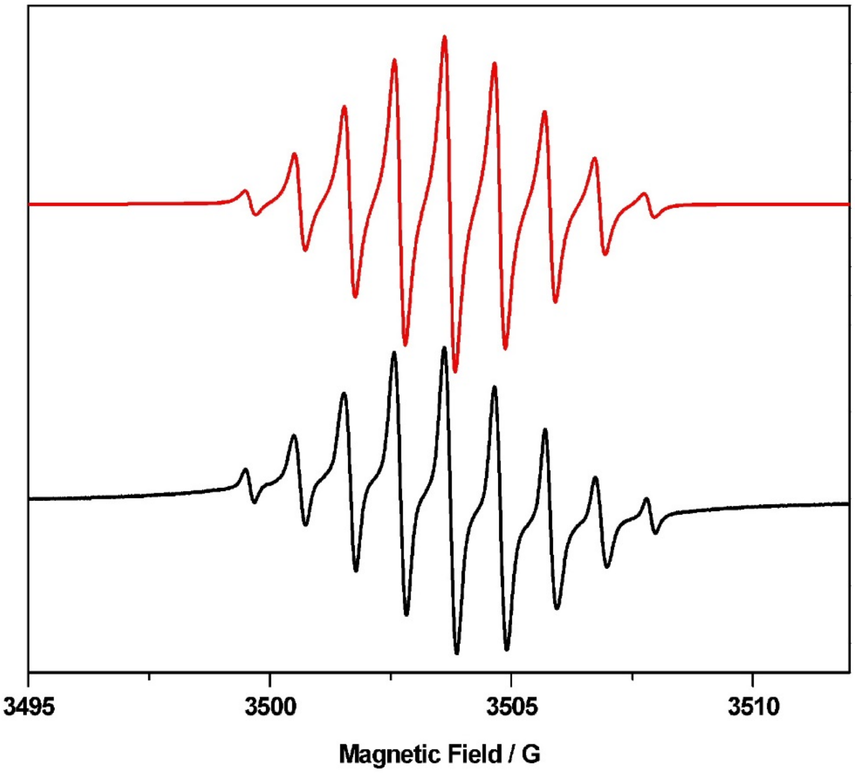

Figure S10 Fluid solution EPR spectrum of $[P3A2Q]^{2-}$ in CH_2Cl_2 containing $[^nBu_4N][BF_4]$ (0.4 M) as supporting electrolyte, at ambient temperature.

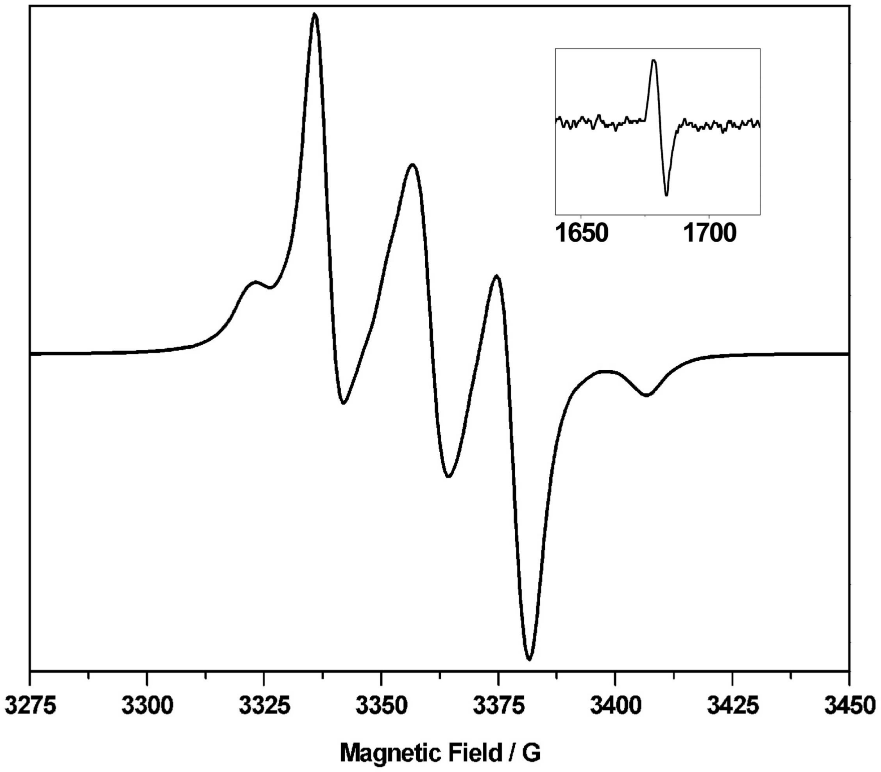

Figure S11 Frozen solution EPR spectrum of $[P3A2Q]^{2-}$ in CH_2Cl_2 containing $[^nBu_4N][BF_4]$ (0.4 M) as supporting electrolyte, at 77 K.

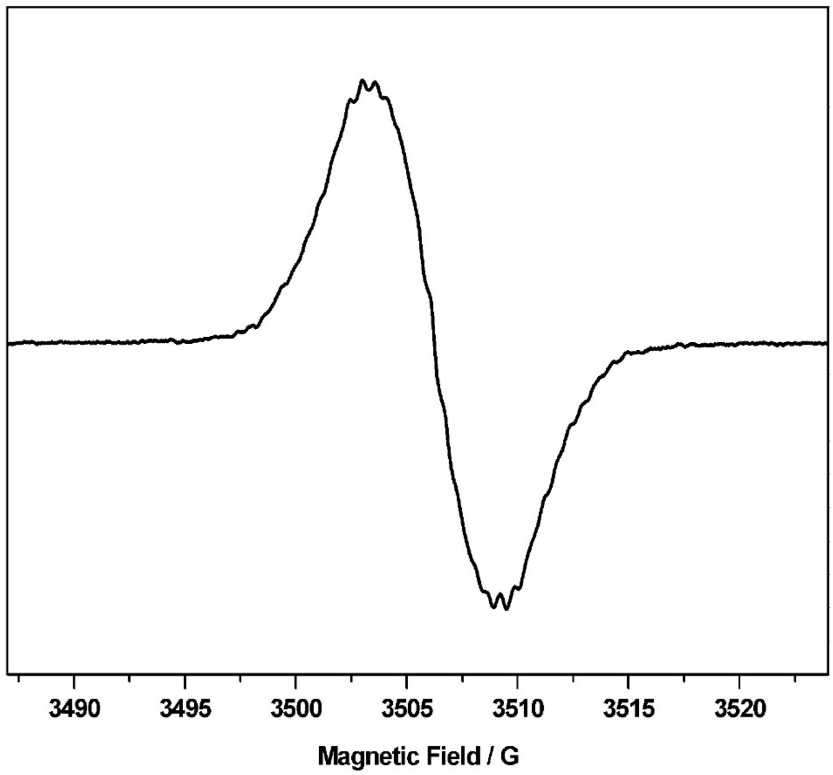

Figure S12 Fluid solution EPR spectrum of $[P4A1Q]^{+}$ in CH_2Cl_2 containing $[^nBu_4N][BF_4]$ (0.4 M) as supporting electrolyte, at ambient temperature.

References

1. S1. Langer, P.; Yang, L.; Pfeiffer, C.R.; Lewis, W.; Champness, N.R. Restricting shuttling in bis(imidazolium)…pillar[5]arene rotaxanes using metal coordination. *Dalton Trans*. **2019**, 48 58-64. [http://dx.doi.org/10.1039/c8dt04096f]
2. S2. Xie, C.; Hu, W.; Hu, W.; Liu, Y.A.; Huo, J.; Li, J.; Jiang, B.; Wen, K. Synthesis of Pillar[n]arene[5−n]quinines via Partial Oxidation of Pillar[5]arene. *Chin. J. Chem*., **2015**, 33, 379-383. [http://dx.doi.org/10.1002/cjoc.201400895]
